# New Estimates of US Civil War mortality from full-census records

**DOI:** 10.1073/pnas.2414919121

**Published:** 2024-11-18

**Authors:** Joan Barceló, Jeffrey L. Jensen, Leonid Peisakhin, Haoyu Zhai

**Affiliations:** ^a^Division of Social Science, New York University-Abu Dhabi, Abu Dhabi, United Arab Emirates

**Keywords:** US Civil War, mortality estimates, methodology

## Abstract

The Civil War was the deadliest conflict in US history. However, incomplete records have made it difficult to estimate the exact death toll both nationally, and especially, at the state level. In this article, we leverage the recently released full count of individual census returns and a sample of linked records across multiple censuses to provide i) the most precise national estimate of excess mortality to date and ii) reliable state-level estimates of excess mortality among native-born white males. Our national estimate is 698,000 Civil War deaths. This is substantially higher than the conventional historical estimate of 618,000 but lower than the most recent estimate of around 750,000 deaths based on a 1% census sample. Leveraging a novel migration-adjusted census comparison method, we document the extent to which the war’s toll was much greater in the Confederate states than in the Union.

The Civil War (1861–1865) was the deadliest conflict in American history. However, there is still disagreement over the exact estimate of lives lost largely because of incompleteness of the Confederate Army’s surviving records. For over a century, the de facto official count was 618,222 total deaths, widely seen to be a gross undercount (1). It was estimated by applying Union Army mortality rates to a rough count of the total number of Confederate soldiers (2). In 2011, J. David Hacker introduced a census-based sex-differential method for estimating excess mortality caused by the war; that study increased the number of dead by more than 20% to over 750,000 (3). This revised figure deservedly received a great deal of scholarly and public attention^*^ and emphasized the deadly toll of what is possibly the most important event in US history (1, 4).

Hacker noted two major limitations to his data and method that we revisit. First, the historical census data were incomplete. The method used a 1% sample of individuals from each of the 1850 to 1880 censuses, which introduced potential for sampling error in the estimates. Second, while the sex-differential census comparison method is effective for generating a national estimate of the death toll where out-of-country migration is negligible, it is not suitable for inferring excess mortality estimates in subnational geographies due to significant levels of intercensal migration across regions, states, and counties. Recently, a full count of individual census returns has become available for 1850–1940 (5), as well as a sample of linked records across multiple censuses that are useful for estimating subnational migration. Using these newly available historical data, published as part of the integrated public use microdata series (IPUMS) (6), we re-estimate the Civil War’s national death toll and introduce a novel census-based method for calculating state-level excess mortality while accounting for cross-border migration.^†^

The sex-differential method for calculating excess mortality compares the observed difference in death rates between military-age male and female native-born whites (ages 10 to 44 in 1860) to the “regular” intersex differences in death rates during the 1850–1860 and 1870–1880 peacetime periods. Subtracting wartime from peacetime mortality produces an estimate of excess mortality due to war; this includes, primarily, soldiers’ and sailors’ deaths but also veterans’ deaths postwar, and deaths of civilians from guerilla fighting. This method, however, is ill-suited for estimating subnational differences in war-related mortality using current residence data given that cross-state migration was sizable in the 1860s. For instance, a Union Army veteran who resided in Indiana at the time of the 1860 Census and was living in a Southern state (e.g., Georgia) in 1870 would erroneously add a Civil War death to the Union tally (and to Indiana’s), and would subtract from the Confederate total (and Georgia’s).

Leveraging these newly available historical data, we introduce a migration-adjusted census comparison method for estimating excess mortality in the Civil War at the subnational level. First, for each state we estimate the *expected* hypothetical peacetime raw death rate in the 1860–1870 census period for each 10-y age cohort of military-age (5 to 44) native-born white males (hereafter, NBWM) by averaging observed death rates for these age groups in the 1850–1860 and 1870–1880 periods.^‡^ Second, we calculate raw *excess mortality* due to the Civil War in each age cohort; this is the difference between expected and observed mortality in the 1860–1870 census decade. Third, using the linked census records, we similarly estimate the expected outmigration rate in 1860–1870 in each age cohort by averaging the observed peacetime rates in 1850–1860 and 1870–1880, and then calculate the excess war-related outmigration rate by subtracting the observed rate from this expected rate. Next, we subtract the excess outmigration rate from the excess mortality rate. This leaves us with a migration-adjusted excess mortality rate. Finally, to turn that rate into a raw count of deaths we multiply it by the 1860 population of NBWM and sum the numbers across all the age cohorts to arrive at a state-level tally. Adding state tallies across the Confederacy and the Union we arrive at bloc-specific excess mortality estimates ( 8).

## New Estimate of Total War-Related Deaths

In Table 1, we present the updated estimate of Civil War deaths using the sex-differential method based on the full census count against Hacker’s estimate from the 1% sample. The count among NBWM is our estimate of excess mortality against the average baseline of regular mortality in the two adjacent census periods. The lower and upper bounds in brackets below each estimate are the excess mortality figures calculated separately from the 1850–1860 and 1870–1880 baselines for regular mortality. Using the full count census records, we estimate that there were nearly 497,000 excess deaths due to the Civil War among military-age NBWM against an estimate of approximately 539,000 using the 1% sample.

**Table 1.** Excess mortality in the US Civil War: sex-differential method with 1% sample vs. full count census data

	1% Sample	Full count
Native-Born White	538,842	496,332
Males (NBWM)	(450,561 to 627,124)	(462,273 to 530,391)
Total Civil War	751,562	698,000
Deaths	(632,115 to 871,009)	(647,439 to 748,561)

To arrive at the total count of war-related deaths, we follow Hacker in adjusting for a recognized 6% undercount in the 1860 census, adding an estimate of excess mortality among foreign-born white males (assuming the same excess death rate as among native-born white males), and 36,000 Union Black soldier deaths.^§^ We estimate the total toll of the Civil War to be around 698,000 deaths. This is about 54,000 deaths or 7.2% lower than the estimate with the 1% sample. Importantly, our estimate has a bounding interval of roughly ± 50,000, which is about half the size of the interval calculated using the 1% sample. Moreover, while the upper estimate with the 1% sample could be as high as 871,000 deaths, our more precise estimates reduce the plausible highest death toll by 122,000 to approximately 749,000.

Our findings confirm that Civil War deaths have long been underestimated by around 13% relative to the death toll of 618,000 popularized in the late 19th century. At the same time, evidence from the full census also suggests that the estimate using the 1% sample overstated the most probable death toll by 7% and the upper bound by 14%. Overall, to the best of our knowledge, the estimated death toll of 698,000 deaths is the most accurate assessment of Civil War mortality to date.

## State-Level Estimates of Civil War Deaths

**Fig. 1.**
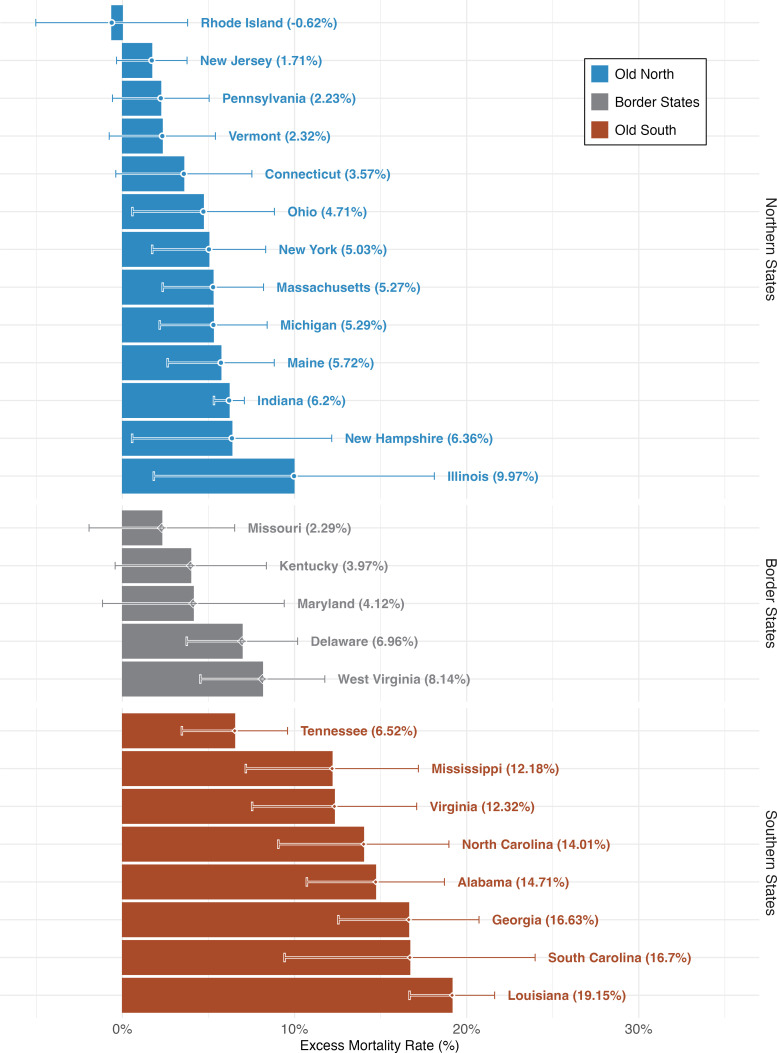
Bar chart showing estimated excess male deaths during the Civil War era among military-aged NBWM. Each bar corresponds to an excess death estimate based on the average baseline; the error bar around the right-hand edge of the bar corresponds to analogous estimates using the regular mortality baselines of 1850–1860 and 1870–1880, respectively.

This report’s main contribution is that we are able to provide reliable estimate of the Civil War’s death toll at the state level using the migration-adjusted census comparison method. One limitation is that our ability to calculate credible estimates is hampered in frontier regions that were settled around the mid-19th century, where census records are especially unreliable. All in all, we are able to accurately estimate the war’s death toll in core states that were settled before 1830 and whose populations comprised 90% of the US’ native-born white fighting-age males. This includes 13 of the 18 nonenslaved Northern states (what we term Old North), 8 of the 11 slaveowning Confederate states (Old South), and the remaining five slaveowning states, including West Virginia, which did not join the Confederacy (Border States). The states in each grouping are listed in Fig. 1.

In Table 2, we present the excess mortality estimates for the three regions: Old North, Border States, and Old South. Combining these migration-method bloc estimates with our national count from the sex-differential method suggests that these regions accounted for 93% of all Civil-War deaths among military-age NBWM (460,032/496,332).^¶^

**Table 2.** Regional excess mortality in the Civil War

	Native-born white males (NBWM, 5 to 44)	Migration-adjusted total deaths	Migration-adjusted mortality rate
Old North	4,669,627	229,803	4.9%
		(155,943 to 303,664)	(3.3% to 6.5%)
Border States	942,787	38,069	4.0%
		(25,456 to 50,682)	(2.7% to 5.4%)
Old South	1,471,203	192,160	13.1%
		(129,269 to 255,051)	(8.8% to 17.3%)
Total	7,083,617	460,032	6.5%
		(310,668 to 609,396)	(4.4% to 8.6%)

The states in each region are shown in Fig. 1.

The difference in death tolls across regions demonstrates powerfully how much deadlier the Civil War was for the Confederacy than the Union. Although the core of the Confederacy had fewer than one-third as many military-age NBWM as the core of the Union, states at the core of the Confederacy suffered almost as many casualties (192,160 deaths in the Old South vs. 229,803 in the Old North). This translates into an excess mortality rate of 13% in the Confederacy against only 5% in the Union.

If we apply the 13% excess mortality rate to the remaining three Confederate states not in our analyses and include excess mortality from the Border States^#^ and foreign-born fighters who fought for the Confederacy then the total estimate of deaths in the Confederacy would approach 300,000. Doing the same for the Union states would bring the death tally there to more than 400,000. Combined, these totals match the national tally of 698,000 deaths reported earlier.

In Fig. 1, we present the state-level estimates of war-related excess mortality among military-age NBWM. This figure further illustrates the disproportionate impact of the war on the Confederacy. While seven of the eight Old South states saw at least 10% of their military-age NBWM killed, only one Northern state, Illinois, experienced comparable mortality. The migration-adjusted method permits estimates of war-related deaths at the age cohort level in each state. When examining excess mortality for the different age cohorts, we find that in most Confederate states around 20 to 33% of NBWM aged 15 to 34 in 1860 perished in the war. In the Northern and border states the same age cohorts suffered mortality of below 10% in all but a handful of states.

## Discussion

Concerns about the accuracy of the US Civil War’s death toll have persisted for over a century due to incomplete Confederate records. The most recent attempt to re-estimate the death toll was based on a 1% census sample. In this report, we leveraged the recently released full-count census records and information linking individuals across multiple censuses to provide i) the most accurate estimate of war-related excess mortality at the national level, and ii) state and bloc-level estimates of the death toll among native-born white males. By our calculation, 698,000 individuals perished as the result of the Civil War. This revises upward the long-held underestimate of 618,000 (+14%) and adjusts downward by 54,000 (−7%) the most recent overestimate from the 1% sample. Our migration-adjusted excess mortality method highlights the war’s disproportionate impact on the Confederacy: Southern states saw an average of 13% of their military-age NBWM die, compared to fewer than 5% in Northern states. Notably, this method can also be used at the county level for more fine-grained analyses.

Due to the lack of quality subnational war mortality estimates, it has been difficult to date to fully assess the long-term impact of the Civil War. Studies that have used the full census records in the much less affected Northern states have shown how children whose father had been killed in the war experienced considerably worse labor outcomes than their unaffected peers (10). It seems reasonable to expect that the enormous mortality in the US South shaped state demographics, impacting families through the death of bread-winners and triggering economic, political, and social legacies for generations. The legacies of war trauma likely had consequential effects on political behavior for both the surviving soldiers and their families. For instance, recent evidence shows that, in Germany, areas that experienced greater soldier mortality in World War I also voted for the Nazi Party in greater numbers a few decades later (11). Our methodology and granular estimates of subnational death toll in the Civil War provide scholars the opportunity to study the war’s true impact in greater depth.

## Data Availability

Data and analysis code files (in R) have been deposited in Harvard Dataverse (https://dataverse.harvard.edu/dataset.xhtml?persistentId=doi:10.7910/DVN/RK7UG3 (8)). Previously published data were used for this work (5, 6).

## Footnotes

^*^See, for instance, this coverage in the *New York Times*: https://www.nytimes.com/2012/04/03/science/civil-war-toll-up-by-20-percent-in-new-estimate.html.

^†^The full-count census data come with their own challenges (7), and the linked census data might be biased toward married individuals with larger families.

^‡^Due to age heaping in the census data we use 10-y age cohorts and start with individuals aged 5 in 1860. We allow for a ±5-y window in reported age when matching unique records across censuses. In addition, we work with the state of residence to capture migration, not state of birth.

^§^We cannot use the census-based sex-differential method to estimate foreign-born and Black war-related deaths and have to rely on standard assumptions instead.

^¶^In contrast, the sex-differential method yields only around 360,000 deaths for these regions, which at 72% of the total number of NBWM deaths is too low.

^#^Historians believe that roughly one-third of Border State soldiers fought for the Confederacy (9).
